# Molecular mediators of the association between child obesity and mental health

**DOI:** 10.3389/fgene.2022.947591

**Published:** 2022-08-31

**Authors:** Evangelos Handakas, Yiwen Xu, Alexa Blair Segal, Maria Carmen Huerta, Kirsty Bowman, Laura D. Howe, Franco Sassi, Oliver Robinson

**Affiliations:** ^1^ Μedical Research Council Centre for Environment and Health, Imperial College London, London, United Kingdom; ^2^ Centre for Health Economics and Policy Innovation, Department of Economics and Public Policy, Imperial College Business School, London, United Kingdom; ^3^ MRC Integrative Epidemiology Unit at the University of Bristol, Bristol, United Kingdom; ^4^ Population Health Sciences, University of Bristol, Bristol, United Kingdom

**Keywords:** child, obesity, depression, multiomics, ALSPAC

## Abstract

Biological mechanisms underlying the association between obesity and depression remain unclear. We investigated the role of metabolites and DNA methylation as mediators of the relationship between childhood obesity and subsequent poor mental health in the English Avon Longitudinal Study of Parents and Children. Obesity was defined according to United Kingdom Growth charts at age 7 years and mental health through the Short Mood and Feelings Questionnaire (SMFQ) completed at age 11 years. Metabolites and DNA methylation were measured by nuclear magnetic resonance spectroscopy and Illumina array in blood at the age of 7 years. The associations between obesity and SMFQ score, as continuous count data or using cut-offs to define depressive symptoms (SMFQ >7) or depression (SMFQ >11), were tested using adjusted Poisson and logistic regression. Candidate metabolite mediators were identified through metabolome-wide association scans for obesity and SMFQ score, correcting for false-discovery rate. Candidate DNA methylation mediators were identified through testing the association of putative BMI-associated CpG sites with SMFQ scores, correcting for look-up false-discovery rate. Mediation by candidate molecular markers was tested. Two-sample Mendelian randomization (MR) analyses were additionally applied to test causal associations of metabolites with depression in independent adult samples. 4,018 and 768 children were included for metabolomics and epigenetics analyses, respectively. Obesity at 7 years was associated with a 14% increase in SMFQ score (95% CI: 1.04, 1.25) and greater odds of depression (OR: 1.46 (95% CI: 0.78, 2.38) at 11 years. Natural indirect effects (mediating pathways) between obesity and depression for tyrosine, leucine and conjugated linoleic acid were 1.06 (95% CI: 1.00, 1.13, proportion mediated (PM): 15%), 1.04 (95% CI: 0.99, 1.10, PM: 9.6%) and 1.06 (95% CI: 1.00, 1.12, PM: 13.9%) respectively. In MR analysis, one unit increase in tyrosine was associated with 0.13 higher log odds of depression (*p* = 0.1). Methylation at cg17128312, located in the FBXW9 gene, had a natural indirect effect of 1.05 (95% CI: 1.01,1.13, PM: 27%) as a mediator of obesity and SMFQ score. Potential biologically plausible mechanisms involving these identified molecular features include neurotransmitter regulation, inflammation, and gut microbiome modulation. These results require replication in further observational and mechanistic studies.

## Introduction

The association between obesity and depression is well established, with bi-directional associations widely reported in observational studies (Milaneschi et al., 2019). Recent Mendelian Randomization (MR) studies have confirmed a causal relationship between obesity and depression (Tyrrell 2019). Poor mental health has been proposed as one mechanism through which obesity during childhood may lead to poorer social and economic outcomes in later life (Segal et al., 2021). It was recently shown in the Avon Longitudinal Study of Parents and Children (ALSPAC) cohort that depressive symptoms mediated approximately 11% of the relationship between body mass index (BMI) and General Certificate of Secondary Education (GCSE) scores in girls, demonstrating the importance of mental health to human capital development (Bowman et al., 2022). Both psycho-social and biological mechanisms appear to be important in the causal chain from obesity to depression. The role of psycho-social mediators such as bullying and social stigma has been relatively well-described in population studies (Gini and Pozzoli, 2009; Moore et al., 2017). However, biological mechanisms may explain a significant proportion of the association between obesity and depression in children, which may have implications for policy interventions to improve mental health.

Proposed biological pathways in the causal pathways between obesity and mental health include alterations to the hypothalamic–pituitary–adrenal (HPA) axis, immuno-inflammatory activation, neuroendocrine regulators of energy metabolism, and the microbiome (Marazziti et al., 2014; Milaneschi et al., 2019). Biological mechanisms may be traced using biomarkers such as DNA methylation, an important regulator of gene expression, and metabolites, small molecules that reflect metabolic alterations. Obesity induces widespread changes in methylation levels across the genome (Wahl et al., 2017; Campanella et al., 2018) that may play a role in mood disorders. Differential methylation of a handful of the same genes involved in inflammatory pathways has been identified across separate studies of obesity and mood disorders (Gharipour et al., 2020). In addition, evidence shows BMI-associated DNA methylation predicts diseases, including T2D and cancers (Wahl et al., 2017). Similarly, as a metabolic disorder, obesity leads to widespread perturbation of the metabolome, which may influence mood. For instance, branched-chain amino acids (BCAAs) may influence brain function by modifying large, neutral amino acid transport at the blood–brain barrier, interfering with neurotransmitter synthesis (Fernstrom, 2005) (Gruenbaum et al., 2019). Recent reviews of the effects of obesity on the epigenome and metabolome in children identified consistent increases in both BCAAs and aromatic amino acids (AAAs) (Handakas et al., 2021) and methylation at multiple CpGs (sites of DNA methylation), albeit with less consistency between studies (Alfano et al., 2021).

In this study, we investigated the role of obesity-related metabolic and epigenetic signatures (measured through nuclear magnetic resonance (NMR) spectroscopy and DNA methylation array, respectively) in the onset of poor mental health in childhood in the ALSPAC cohort. We aimed to test the role of metabolites and differentially methylated CpGs as mediators of the relationship between childhood obesity and subsequent poor mental health or depression, assessed through the Short Mood and Feelings Questionnaire (SMFQ).

## Materials and methods

### Study population

The study population included participants from ALSPAC. Initially, the ALSPAC study recruited 14,541 women living in Avon, England, with an expected delivery date between 1 April 1991 and 31 December 1992 (Boyd et al., 2013; Fraser et al., 2013). Participants have been followed up with questionnaires and clinical measures at regular intervals, providing lifestyle, socioeconomic, behavioral and biological data. The ALSPAC study website contains details of all the data that is available through a data search tool (http://www.bris.ac.uk/alspac/researchers/data-access/data-dictionary/). A total of 4,018 had anthropometric data available at 7 years (early childhood) and the SMFQ available at 11 years. The timepoints were chosen to maintain the temporal relationship with the molecular data measured at 7 years and preceding the mental health assessment. A flowchart of individuals that were considered eligible for the analysis is shown in Supporting Information S1 (Supplementary Figure S1).

### Obesity classifications

Child height and weight were measured at a clinic visit at age 7. Obesity was defined as a BMI at or above the 95th percentile of the British 1990 population growth reference (Cole et al., 1995). The Sitar package in R was used to classify the study population as either with obesity or without obesity (Cole and Cole, 2021).

### Mental health classifications

Mental health and depression in children were assessed in the ALSPAC cohort at a clinic visit at age 11 years, using the SMFQ (Ancold and Stephen, 1995). This questionnaire consists of a 13-item checklist of depression symptoms experienced in the previous 2 weeks. The children completed the SMFQ at research clinics with trained interviewers. Each item is rated on a 3-point scale: zero (not true), one (sometimes true) or two (true), giving a total score of 0–26. Higher scores suggest higher depression symptoms. To increase power in our analysis, we primarily used the SMFQ score as a count variable to assess the percentage change in score across the whole score distribution (Delmastro and Zamariola, 2020). In the larger subset with metabolomic data available, we also used pre-defined cut-offs to assess consistency across different definitions commonly used: Children with “depressive symptoms” were defined as those with scores over 7 (Ancold and Stephen, 1995) and “depression” cases were defined as those with scores over 11 (Turner et al., 2014).

### Covariates

Maternal education was classified into three categories according to mothers’ highest educational achievement (Handakas et al.): 1) low: Ordinary- (O-) level, educational qualifications generally obtained at 16 years of age, Certificate of Secondary Education (CSE); 2) intermediate: Advanced- (A-) level; 3) high: university degree and above. Family income at 7 years was classified into two categories: 1) low/medium: <£400 per week, and 2) high: ≥£400. Physical activity at 5 years was classified into two categories based on the frequency of going to the park or playground compared with other children: 1) less often and 2) similarly or more often than other children. Birth weight was obtained from notifications or clinical records. Maternal pre-pregnancy BMI was calculated from self-reported weight and height at the 12th week of gestation.

### Metabolomics

Metabolomic profiling was carried out using ^1^H nuclear magnetic resonance spectroscopy on fasting plasma samples from 4,018 individuals of the ALSPAC cohort at 7 years. This molecular signature of systemic metabolism consists of 230 metabolic traits (Supporting Information S2, Codebook). The platform provided quantification of 14 lipoprotein subclasses (particle concentration, lipid concentrations and composition), fatty acids and fatty acid composition, ketone bodies, amino acids, gluconeogenesis-related metabolic traits and glycolysis and gluconeogenesis-related metabolites (Soininen et al., 2009; Kujala et al., 2013). Details related to the used platform have been published previously (Soininen et al., 2009). Data were log-transformed and scaled before analysis.

### Epigenetic data

The Accessible Resource for Integrated Epigenomics Studies (ARIES) is a sub-study drawn from the ALSPAC mother-child cohort that consists of 1,018 mother-child pairs (Relton et al., 2015a) and includes 5469 DNA methylation profiles obtained from umbilical cord blood at birth or at clinic visits at several time points after birth. After DNA extraction, samples were bisulphite converted using the Zymo EZ DNA Methylation ™ kit (Zymo, Irvine, CA, United States). Genome-wide methylation was measured using the Illumina Infinium HumanMethylation450 (HM450) Bead-Chip. The arrays were scanned using an Illumina iScan, and an initial quality review was assessed using GenomeStudio. The preprocessing and normalization of data were carried out using the meffil R package (Min et al., 2018). The LIMS also reported quality control (QC) metrics from the standard control probes on the Illumina 450 K array for each sample (Relton et al., 2015b). Low-quality profiles were removed from further processing. The remaining profiles were normalized using the Functional Normalization algorithm with the first 10 control probe principal components (Fortin et al., 2014). For additional QC steps, we cross-checked candidate CpGs of arrays using a list of cross-reactive probes and polymorphic CpGs (Chen et al., 2013). Moreover, probes as well as samples with more than 5% missing values were removed from the dataset. Finally, we converted the beta values to M-values using the logit transformation to avoid severe heteroscedasticity for highly methylated or unmethylated CpG sites as well as to improve performance in terms of Detection Rate (DR) (Du et al., 2010).

### Methylation risk score

**FIGURE 1:**
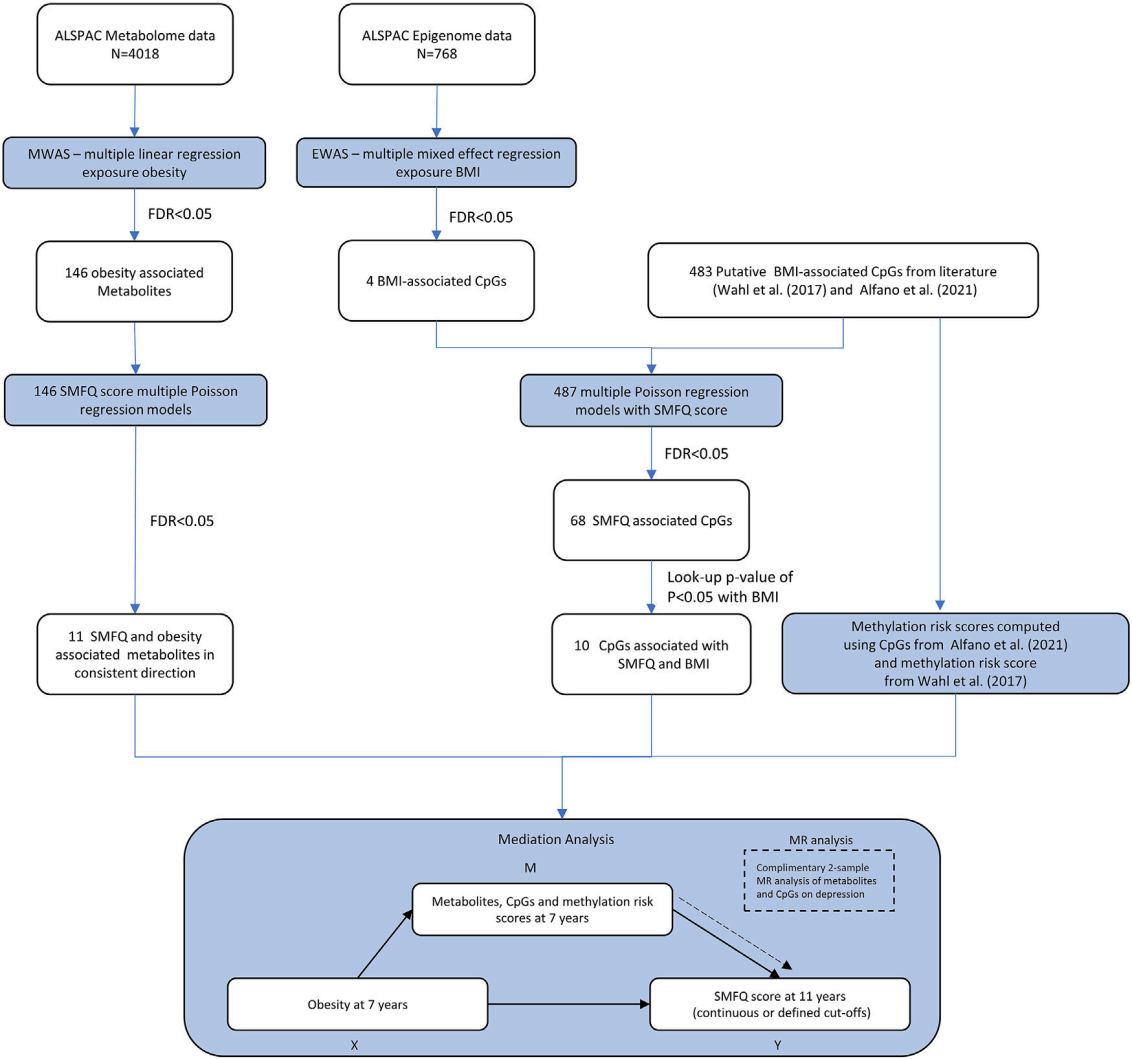
Overview of the analysis and methodological workflow. Mediation analysis is a directed acyclic graph analytical framework of the hypothetical causal relationship between an exposure (X) and an outcome (Y) through a mediator (M). The blue rectangles are the statical analysis steps.

To combine information across loci, we calculated two weighted methylation risk scores (MRS) as the sum of the standardized methylation values. The first risk score was calculated as weighted by marker-specific effect size MRS from the Wahl et al. (2017) study in adults. The second was calculated using CpGs from the Alfano et al. (2021) systematic review, respectively (Figure 1). We calculated the weights for the Alfano et al. (2021) MRS using ALSPAC data and the conducting elastic net using the R Glmnet package (Friedman et al., 2010). Elastic net model parameters, alpha (that defines mixing between lasso and ridge penalties) and lambda (overall strength of penalty), were found following 10-fold cross-validation. The 10-fold routine was performed on the training set (random 80% of the total observations) for each model to estimate elastic net model parameters, alpha and lambda, similar to the methodology of Robinson et al. (2020). The model performance was evaluated on the relevant test set (remaining 20% of the total observations) using a mean square error classifier.

### Data imputation

To maximize power and potentially reduce bias in our analysis, we applied a multivariable multiple imputation procedure to impute the missing values of covariates. To further ensure the results’ power and reduce potential bias, proportions and patterns of missingness were checked before imputation. Normalized root-mean-square error and out-of-bag error were used as the evaluation metrics of imputation for the set of continuous and categorical variables, respectively. The subsets for epigenetic and metabolomic data were imputed separately. Imputation was carried out based on the multiple chained equations method with the R package mice (Buuren and Groothuis-Oudshoorn, 2010) with the assumption that data were missing at random. First, we performed 100 imputations by 1,000 chains of regression, and then we applied Rubin’s rule (Rubin, 2004) for combining the separate estimates and standard errors from the analytical models performed on each of the 100 imputed datasets.

The metabolomics data were imputed using the random forest (RF) (Breiman, 2001) approach. This is a non-parametric method that allows for interactive and non-linear (regression) effects and has a high performance ability in imputing missing values at random and not at random (Wei et al., 2018). The imputation was carried out using the missForest R package (Stekhoven and Bühlmann, 2012) with default parameter settings.

### Statistical analysis

Logistic regression was used to investigate the association between obesity at 7 years and depression or the presence of depressive symptoms at 11 years (total effect). Poisson regression was used to model the association between obesity at 7 years and SMFQ scores as a continuous (count) variable (total effect). Both models were adjusted for sex, age, mother’s pre-pregnancy BMI, birth weight, physical activity at age 5, maternal education, and family income, selected based on literature and univariate associations (*t*-test and chi-squared tests) in the dataset.

We applied a Metabolome-Wide Association Study (MWAS) to investigate the associations between obesity at 7 years and metabolomics at 7 years and the associations between metabolomics at 7 years and the depression outcomes at 11 years. Multiple logistic regression models were used to analyze associations between metabolites and depressive symptoms of depression, and multiple Poisson regression models were used to analyze associations between metabolites and the SMFQ score. The models were adjusted for the same covariates aforementioned.

Additionally, an Epigenome-Wide Association Study (EWAS) was applied to investigate the association between DNA methylation and BMI using multiple linear mixed-effects models at the age of 7. The model was adjusted for age, sex, and BMI as fixed effects, and position on chip (“pos”) and beadchip (“slide”) were accounted for through random effects. Additionally, Houseman-estimated cell proportions (Houseman et al., 2012) (B cells, CD8^+^ T cells, CD4^+^ T cells, granulocytes, NK cells, and monocytes) were used as fixed effects to adjust for cellular heterogeneity in blood DNA. The methylation beta values were expressed as a logit transformation of the percentage of the methylation (Du et al., 2010). To account for multiple testing, we applied a false discovery rate (FDR) using a cut-off of 5%.

Then, we explored the associations of these CpGs with SMFQ score at 11 years old by filtering the EWAS results by a look-up list of putative BMI-associated CpGs. These included 184 CpGs from the study of Wahl et al. (2017) in adults (Supporting information S2, Supplementary Table S1), and 299 CpGs reported at least once to be associated with adiposity in children from a recent review by Alfano et al. (2021) (Supporting information S2, Supplementary Table S2). Starting from the look-up list of 483 CpGs plus the four CpGs identified in the applied EWAS, we investigated associations with SMFQ score using multiple adjusted Poisson models. CpGs were considered significant using an FDR 5% correction on this CpG list.

Finally, we conducted a sensitivity analysis stratified by sex for both the MWAS and EWAS models.

The analysis workflow is available in Figure 1. The analysis was carried out in the R programming language and these R libraries are presented in Supporting information S1.

### Mediation analysis

Mediation analysis was conducted to assess whether the effect of obesity at age seven on depressive symptoms of depression or SMFQ score at age 11 was mediated by metabolome and DNA methylation profile. In brief, the aim of the mediation analysis was to quantify the effect of X on Y mediated by M (X → M → Y) (natural indirect effect, NIE) and the effect of X on Y that does not operate through the mediator (X → Y): controlled direct effect (CDE), Figure 1.

We used the imputation approach (Vansteelandt et al., 2012) to estimate the conditional natural direct effect (NDE) (not mediated by metabolites and DNA methylation sites) and the conditional natural indirect effect (NIE) (mediated by metabolites and DNA methylation sites) of obesity on depressive symptoms of depression, using the medflex package in R (Steen et al., 2017). We modelled the effect of obesity on SMFQ as a continuous count variable using Poisson regression.

The mediator and outcome models were adjusted for sex, age, mother’s pre-pregnancy BMI, birth weight, physical activity at age 5, maternal education, and family income. For all the mediation models, confidence intervals of 95% (95% CI) were calculated by a non-parametric bootstrap with 1,000 replications. Bootstrapping was used for testing the indirect effect because it does not assume normality in sampling distribution (Hayes and Rockwood, 2017). The mediation proportion was calculated using the following formula: Proportion Mediated = NIE/(NIE + NDE) (Rijnhart et al., 2019).

Similarly, we carried out a sensitivity analysis on the mediation models by applying stratification by sex.

### Two-sample MR of molecular features and depression

MR analyses were performed to assess the causal influence of molecular features, identified as potential mediators in the observational analyses, on depression in independent adult samples (Figure 1).

Two-sample MR was conducted using the TwoSample R package (Hemani et al., 2018). For the metabolites, genetic instruments were downloaded through the IEU Open GWAS Project (Elsworth et al., 2020). Summary statistics for SNPs associated with metabolites came from the most recent United Kingdom Biobank analysis (IEU Open GWAS Batch ID: “met-d”), while instruments for depression were obtained from analysis in the round four data release from the Finnish FinnGen Biobank ((IEU Open GWAS ID: “finn-a-F5_DEPRESSIO”) https://www.finngen.fi/en).

For CpGs identified in this study, we downloaded available summary statistics from http://mqtldb.godmc.org.uk/. SNPs in linkage disequilibrium were pruned using the European reference panel and default parameters using the “clump_data” function. In the CpG analysis, we used as the instrument for depression the summary statistics from the most recent United Kingdom Biobank analysis (IEU Open GWAS ID: “ukb-d-F5_DEPRESSIO”) as the largest available and non-overlapping study.

## Results

### Study population of metabolomic analysis

**FIGURE 2:**
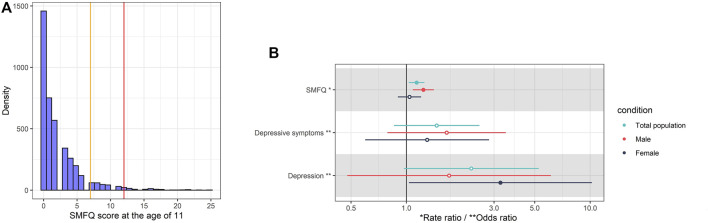
**(A)** Histogram of SMFQ score at 11 years. The vertical orange and red lines are the cut-offs of depressive symptoms (SMFQ>7) and depression (SMFQ>11), respectively. **(B)** Rate ratio per standard deviation (95% CI) of obesity at 7 years (exposure variable) for SMFQ score (outcome), and odds ratio of obesity at 7 years (exposure variable) for depressive symptoms (SMFQ>7) (outcome), and depression (SMFQ>11) at 11 years (outcome). The model is adjusted for sex and age, physical activity at age 5, mother’s age at birth, birthweight, mother’s pre-pregnancy BMI, average weekly family income at age 7, and mother’s highest education qualification. The error Bars show 95% confidence intervals (calculated through parametric methods).

After the removal of children without metabolomic data at 7 years and those with a SMFQ score at 11 years, this study included 4,018 children (Supporting information S2, Supplementary Figure S1). Table 1 includes the participant information for subsamples included in the metabolomic and epigenetic analyses. Figure 2A shows the distribution of SMFQ scores, which was left-skewed (Figure 2A). 85 children were classified as being depressed, and 262 children were classified as having depressive symptoms (Table 1).

**TABLE 1:** Descriptive statistics in study population with metabolomic and epigenetic data available.

	Metabolomic analysis population	Epigenetic analysis population
	(N = 4,018)	(N = 768)
Child age (years)		
Mean (SD)	7.51 (0.303)	7.45 (0.127)
Sex of child		
Male	2037 (50.7%)	382 (49.7%)
Female	1981 (49.3%)	386 (50.3%)
Child weight (kg)		
Mean (SD)	25.7 (4.41)	26.0 (4.48)
Child height (cm)		
Mean (SD)	126 (5.55)	126 (5.17)
Children with obesity		
Without obesity	3,852 (95.9%)	725 (94.4%)
With obesity	166 (4.1%)	43 (5.6%)
Age of mother during pregnancy (3rd month)		
Mean (SD)	29.5 (4.25)	29.8 (4.32)
Maternal pregnancy BMI (kg/m2)		
Mean (SD)	22.8 (3.32)	22.7 (3.45)
Family income		
< £400	2065 (51.4%)	346 (45.1%)
> £400	1953 (48.6%)	422 (54.9%)
Maternal education		
O level/CSE/none/vocational	2,593 (64.5%)	491 (63.9%)
A level/	782 (18.1%)	175 (22.8%)
Degree and above	643 (17.3%)	102 (13.3%)
Frequency of going to park or playground compared with other children at 5		
Less	807 (20.1%)	154 (20.1%)
Similar or more	3,211 (79.9%)	614 (79.9%)
Depression score over 7		
Case	262 (6.5%)	39 (5.1%)
Control	3,756 (93.5%)	729 (94.9%)
Depression score over 11		
Case	85 (2.1%)	17 (2.2%)
Control	3,933 (97.9%)	751 (97.8%)

SD: Standard deviation. Obesity classification based on the British 1990 population growth reference (Cole et al., 1995).

### Associations between obesity and mental health

In adjusted analyses, obesity was associated with a 14% increase in SMFQ score (rate ratio (RR): 1.14, 95% CI: 1.04, 1.25, *p* = 0.01). Using defined screening cut-offs of SMFQ score, children with obesity had a 46% higher risk (Odds Ratio (OR): 1.46 95% CI: 0.78, 2.38, *p* = 0.16) of depressive symptoms (SMFQ >7) and a 3-fold higher risk (OR: 2.25, 95% CI: 0.96, 5.24, *p* = 0.06) of depression (SMFQ >11) at age 11 than their peers without obesity. (Figure 2B).

Stratifying by sex, we observed a stronger effect with SMFQ score and depressive symptoms in boys, but a stronger effect with depression in girls. Obesity was associated with a 23% increase in SMFQ score in boys (OR: 1.23, 95% CI: 1.08, 1.41, *p* = 0.02) while obesity was associated with a four-fold increased risk of depression in girls (OR: 3.24, 95% CI: 1.04, 10.21, *p* = 0.04) (Figure 2B).

### Metabolite associations with obesity

We examined the associations between obesity and metabolites measured at 7 years using an MWAS approach. As expected, obesity had a strong influence on the metabolome, with 146 metabolic measures associated with obesity after correction for 5% FDR (Supporting Information S2, Supplementary Table S3). Obesity was positively associated with amino acids (tyrosine, phenylalanine, isoleucine, leucine, and valine), fatty acids, glycoprotein acetyls, apolipoprotein B, and small, medium, and large low-density lipoproteins (LDL) and very low-density lipoproteins (VLDL) measures. Obesity was negatively associated with high-density lipoprotein (HDL) measures, apolipoprotein A, degree of unsaturation of fatty acids, lactate, citrate, and glycine.

### Metabolite associations with mental health

**FIGURE 3:**
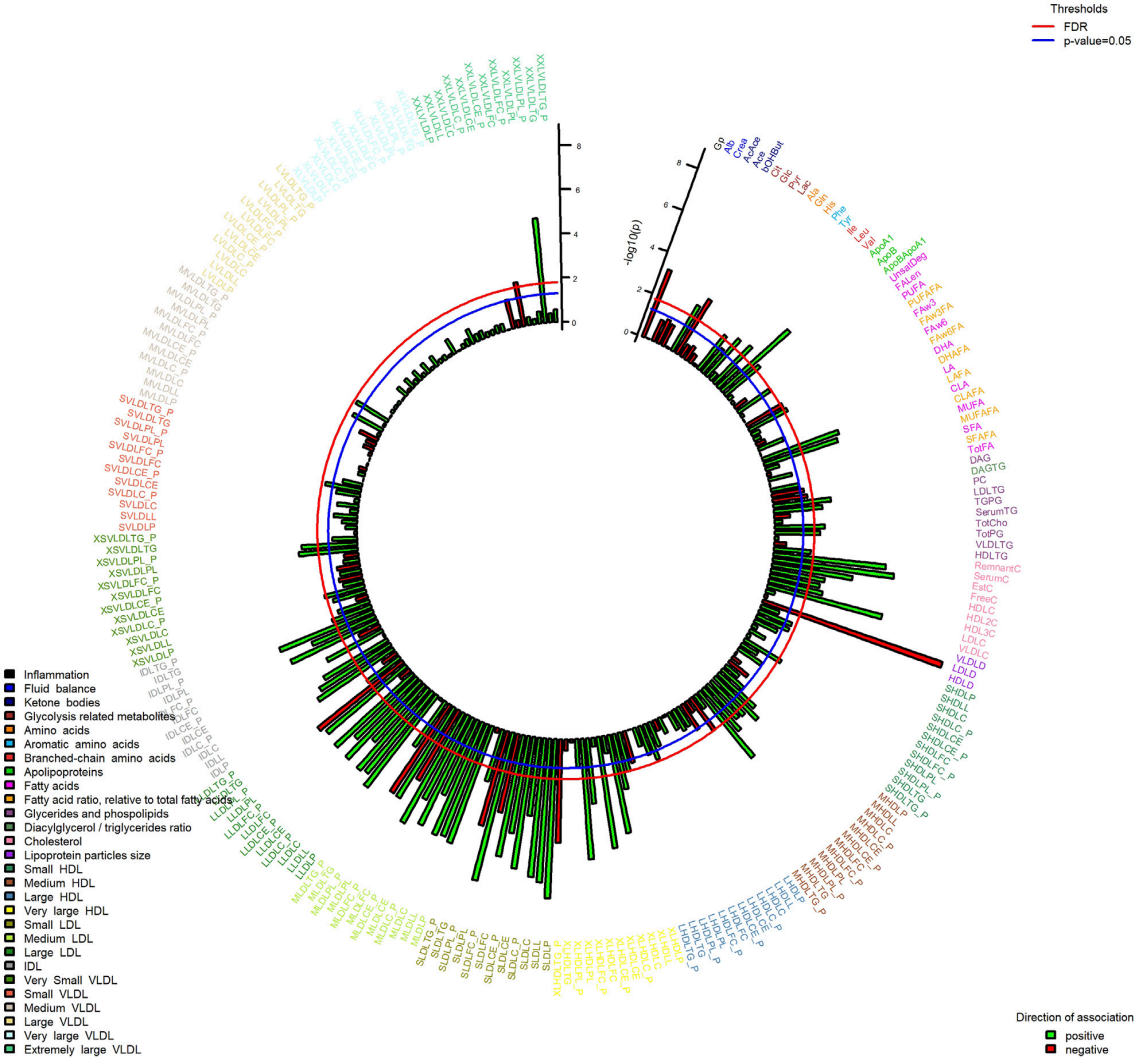
Associations of metabolic measures at 7 years SMFQ score at 11 years (outcome). Bars show strength of association (-log10 *p*-value), colored by direction of effect. Model is adjusted for sex and age of child, physical activity at age 5, mother’s age at birth, birthweight, mother’s pre-pregnancy BMI, average weekly family income at 7, mother’s highest education qualification. FDR at 5%. The abbreviation of the metabolites is available in the Codebook (Supporting Information S2).

In MWAS analysis (Figure 3), we observed associations passing FDR 5% with SMFQ score for 83 metabolites, including lower mean diameter of low-density lipoproteins, glycoprotein acetyls, and glucose and higher levels of histidine, leucine, tyrosine, citrate, apolipoproteins, conjugated linoleic acid, and various lipoprotein measures (Figure 3, Supporting Information S2, Supplementary Table S3). No associations with metabolic measures passed FDR correction for depressive symptoms of depression, although the directions of effect were similar to those with the SMFQ score (Supporting Information S2, Supplementary Table S3).

### Mediation of obesity and mental health by metabolites

To identify candidate metabolites that may play a mediating role between obesity and mental health, we filtered the metabolites by those associated with both obesity and SMFQ score after FDR correction and that had consistent directions of effect. Eleven candidate metabolites were identified as shown in Table 2.

**TABLE 2:** Metabolites simultaneously associated with obesity at 7 years and with SMFQ score at 11, with consistent directions of effect.

	Associations with obesity		Associations with SMFQ score		Associations depressive symptoms (SMFQ >7)		Associations with depression (SMFQ >11)	
Metabolite	Odds ratio	*p*-value	Rate ratio	*p*-value	Odds ratio	*p*-value	Odds ratio	*p*-value
Tyr	1.399 (1.219,1.605)	3.86E-06	1.029 (1.008,1.05)	5.81E-03	1.147 (1.018,1.291)	1.78E-06	1.276 (1.025,1.588)	2.94E-02
Leu	1.395 (1.216,1.6)	4.38E-06	1.03 (1.009,1.052)	4.34E-03	1.101 (0.975,1.243)	2.06E-06	1.24 (0.993,1.548)	5.80E-02
ApoB	1.733 (1.51,1.989)	1.71E-14	1.028 (1.007,1.05)	9.06E-03	1.024 (0.901,1.162)	5.04E-15	0.977 (0.762,1.253)	8.56E-01
CLA	1.368 (1.216,1.539)	4.15E-07	1.038 (1.018,1.059)	2.19E-04	1.124 (1.005,1.257)	1.77E-07	1.083 (0.861,1.364)	4.96E-01
CLAFA	1.282 (1.139,1.443)	7.75E-05	1.037 (1.017,1.058)	3.17E-04	1.128 (1.011,1.258)	3.81E-05	1.103 (0.882,1.38)	3.90E-01
SLDLTG	1.201 (1.049,1.375)	1.35E-02	1.031 (1.01,1.052)	3.72E-03	1.055 (0.934,1.192)	8.18E-03	1.23 (1.003,1.509)	4.69E-02
MLDLPL	1.25 (1.07,1.46)	8.47E-03	1.047 (1.025,1.069)	1.58E-05	1.075 (0.947,1.22)	4.90E-03	1.098 (0.858,1.406)	4.57E-01
LLDLCE_P	1.246 (1.037,1.497)	3.09E-02	1.043 (1.021,1.066)	1.28E-04	1.089 (0.951,1.248)	1.91E-02	1.022 (0.792,1.318)	8.69E-01
IDLCE	1.193 (1.021,1.393)	4.14E-02	1.034 (1.013,1.056)	1.47E-03	1.043 (0.919,1.183)	2.59E-02	0.971 (0.755,1.249)	8.17E-01
XSVLDLL	1.429 (1.233,1.656)	4.38E-06	1.027 (1.005,1.049)	1.35E-02	1.031 (0.908,1.171)	2.04E-06	0.933 (0.723,1.205)	5.97E-01
XSVLDLPL	1.189 (1.016,1.391)	4.87E-02	1.033 (1.012,1.055)	2.12E-03	1.047 (0.922,1.188)	3.11E-02	1.05 (0.818,1.347)	7.02E-01

*Logistic and Poisson regression odds ratio per standard deviation (95% CI) for obesity at seven and year SMFQ at 11 years. Model is adjusted for sex, age of mother at birth, birthweight, mother’s pre-pregnancy BMI, average weekly family income at 7, mother’s highest education qualification. Where ApoB: Apolipoprotein B, CLA: Conjugated linoleic acid, CLAFA: Conjugated linoleic acid (%), IDLCE: Cholesteryl esters in IDL, Leu: Leucine, LLDLCE_P: Cholesteryl esters to total lipids ratio in large LDL, MLDLPL: Phospholipids in medium LDL, SLDLTG: Triglycerides in small LDL, Tyr: Tyrosine, XSVLDLL: Total lipids in very small VLDL, XSVLDLPL: Phospholipids in very small VLDL.

**FIGURE 4:**
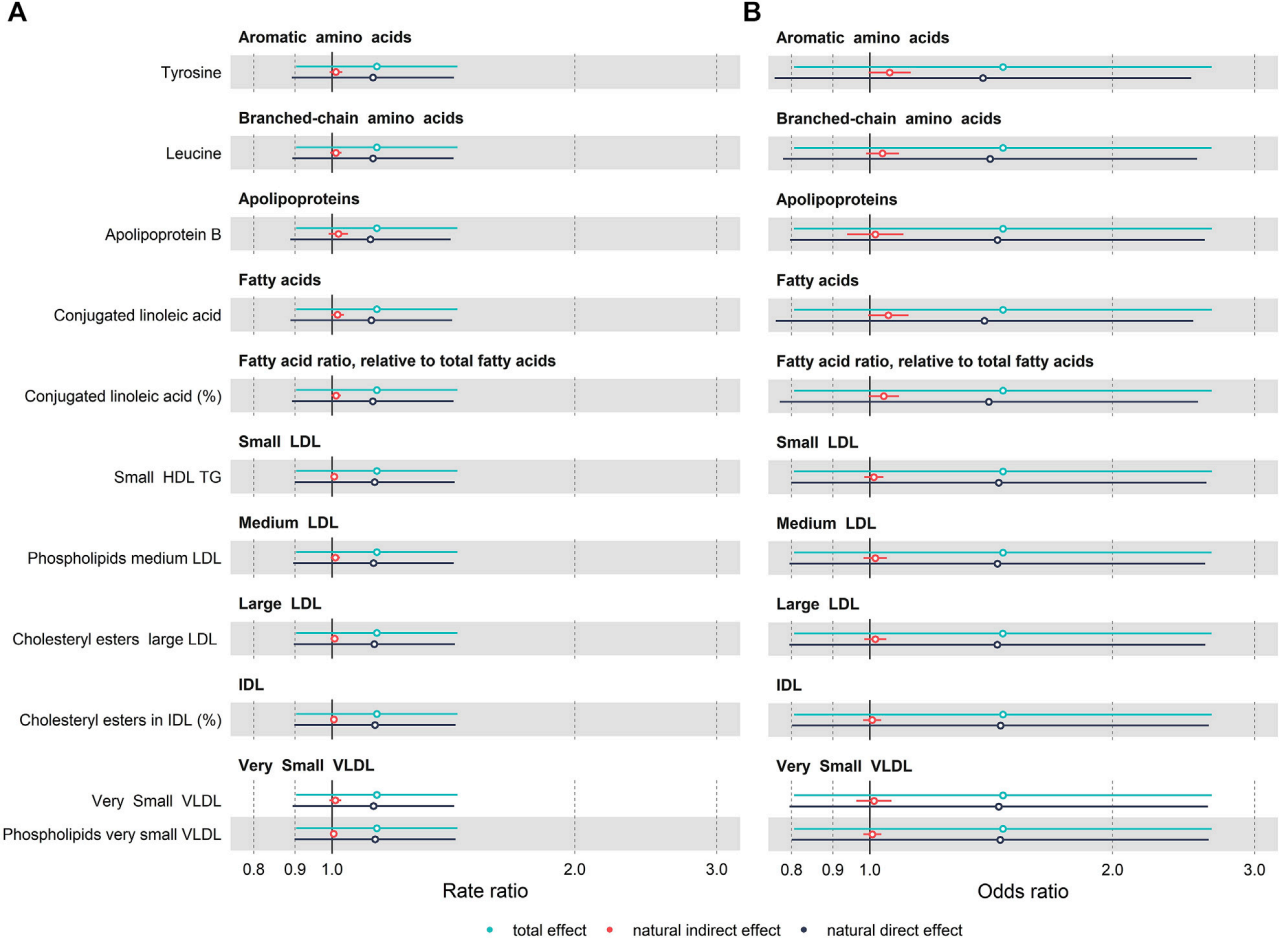
Forest plot of mediation analysis for mood related outcomes, **(A)** SMFQ score and **(B)** depressive symptoms (SMFQ score> 7), across 11 metabolic compounds. Models are adjusted for sex and age of child, physical activity at the age of 5 years, age of mother at birth, birthweight, mother’s pre-pregnancy BMI, average weekly family income at 7, mother’s highest education qualification. The rate and odds ratio are presented as dots. Bars show 95%CI calculated based on a bootstrapping approach (1,000 replications).

We tested for evidence of mediation by these metabolites of the relationship between obesity at 7 years and SMFQ score at 11 years using a counterfactual framework. As shown in Figure 4 and Supplementary Table S4 (Supporting Information S1), most NIEs were positive, suggesting potential mediation, although for many of the candidate metabolites, effects were small with confidence intervals of the NIEs overlapping 1. The largest effect sizes were noted for tyrosine with a NIE for the SMFQ score of 1.11 (95% CI:1.00, 1.27, Proportion Mediated (PM): 12.5%), For leucine, we observed slightly smaller effects with a NIE for the SMFQ score of 1.08 (95% CI: 0.99, 1.19, PM: 9.3%). Additionally, for conjugated linoleic acid (CLA), we noted that for the SMFQ score the NIE was 1.03 (95% CI: 0.94,1.119, PM: 4.1%). Similar effects were observed when CLA was expressed as a ratio to total fatty acids.

A similar overall pattern was observed using the cut-off to define depressive symptoms (SMFQ>7): for tyrosine, the NIE for the depressive symptoms of 1.01 (95% CI: 0.99, 1.03, PM: 8.9%); for leucine, the NIE was 1.01 (95% CI: 1.00, 1.03, PM: 8.7%); and for CLA, the NIE was 1.02 (95% CI: 1.00, 1.04, PM: 12.5%) (Figure 4). Results using the cut-off to define depression (SMFQ>11) are given in Supporting Information S2, Supplementary Table S4, and follow a similar pattern.

### Analysis by sex

The results of MWAS, stratifying by sex, showed that in the case of boys, 94 metabolites–including histidine, leucine, tyrosine, citrate, apolipoproteins, conjugated linoleic acid and various lipoprotein measures–were associated with SMFQ score after controlling the FDR at 5% (Supporting Information S2, Supplementary Table S5). In the case of girls, we found that two metabolites (very large HDL and lipoprotein particle size) were associated with the SMFQ score after controlling the FDR at 5%. In the mediation analysis, stratified by sex, we observed generally stronger mediation by tyrosine for boys compared to girls (Supporting Information S1, Supplementary Figures S3, S4, S5 and Supporting Information S2, Supplementary Tables S7, S8).

### Study population of epigenetic analysis

The population with DNA methylation and outcome data included 764 children (Table 1). The low number of children that would be classified as depressed or having depressive symptoms using a binary classification in this subset precluded analyses on these outcomes (Table 1). We therefore only used the SMFQ score as a continuous variable in the epigenetic analysis. In an adjusted analysis in the subset population with epigenetic data available, we found that obesity was associated with a 20% increase in SMFQ score (Rate Ratio (RR): 1.20, 95% CI: 0.98, 1.46). Although the effect size was similar to the larger subset with metabolomic data available, the confidence intervals overlapped 1. Similar results were obtained when stratified by sex: the RR was 1.29 (95% CI: 0.96, 1.74) in boys and 1.16 (95% CI: 0.89, 1.52) in girls.

### Identification of CpGs for mediation analysis

To select CpGs that may mediate this association, we first performed a genome-wide EWAS for BMI at 7 years. The EWAS analysis showed that cg07462932, cg16332631, cg26271891 and cg26224499 located in the SEPT7, RPS6KA4, ZNF385B and TIMM44 genes respectively were associated with child BMI, after FDR of 5% correction (at genome-level, Table 3, Supporting Information S1, Supplementary Figure S5, Supporting Information S2, Supplementary Table S9).

**TABLE 3:** Ten strongest associations between DNA methylation site (CpGs) and BMI. The model is adjusted for age, sex, CD4T, Bcell, CD8T, Gran, Mono, NK as fixed effect and chip and position bead array as random effect.

Number	CpG	Estimate	Std error^a^	*p*-value	FDR	Chr^b^	UCSC^c^ RefGene name	UCSC RefGene group	Position
1	cg07462932	0.023	0.004	6.37E-08	1.58E-02	chr7	SEPT7	TSS200; TSS200	35840534
2	cg16332631	-0.023	0.004	1.17E-07	1.58E-02	chr11	RPS6KA4	TSS1500	64126102
3	cg26271891	0.027	0.005	1.31E-07	1.58E-02	chr2	ZNF385B; MIR1258	TSS200; TSS1500	180726249
4	cg26224499	0.015	0.003	1.46E-07	1.58E-02	chr19	TIMM44	TSS200	8008578
5	cg25627403	0.024	0.005	7.72E-07	6.70E-02	chr19	HNRNPUL1; HNRNPUL1	TSS1500; 5′UTR	41769009
6	cg26120617	0.023	0.005	1.55E-06	1.09E-01	chr12	NCAPD2; MRPL51	TSS200; TSS1500	6603256
7	cg15592690	0.021	0.004	1.98E-06	1.09E-01	chr18	IMPA2	TSS200	11981389
8	cg04719491	0.026	0.005	2.01E-06	1.09E-01	chr8	HAS2; HAS2AS	TSS1500; Body	122654045
9	cg21845817	0.015	0.003	3.03E-06	1.38E-01	chr2	FAHD2A; FAHD2A	5′UTR; 1stExon	96068451
10	cg21088514	0.027	0.006	3.18E-06	1.38E-01	chr21	KCNE1	TSS1500	35884376

^a^Standard error.

b Chromosom.

c *UCSC:* university of california, Santa Cruz.

Considering the low statistical power to identify BMI-associated CpGs from within our own dataset, we additionally selected putative BMI-associated CpGs from a look-up list. This included 184 CpGs from the study of Wahl et al. (2017) for adults and 276 CpGs reported to be associated with adiposity in children in at least one study from a recent review (Alfano et al., 2021).

**FIGURE 5:**
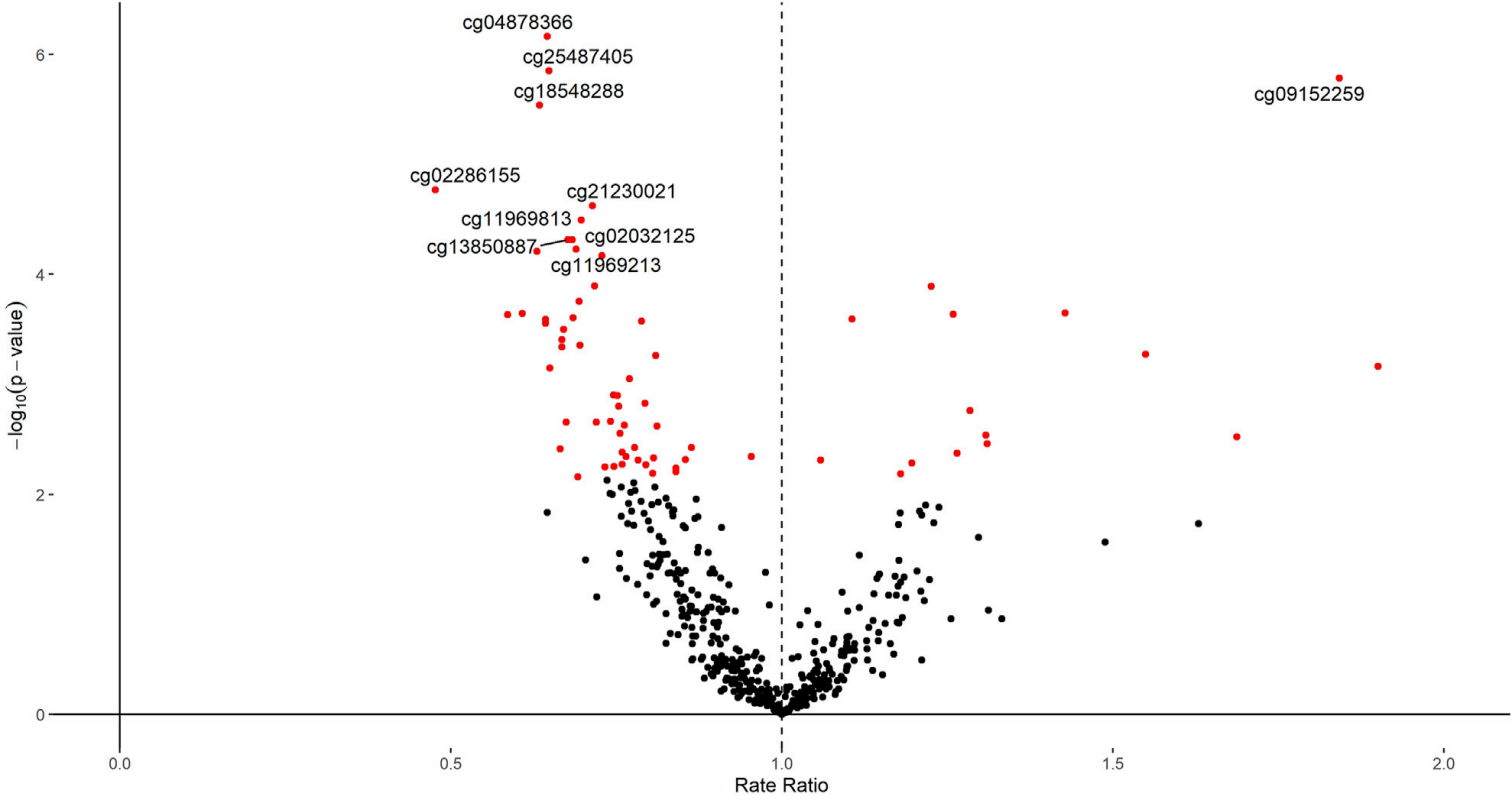
Volcano plot of the *p*-value and *β* coefficient for the association between 483 putative BMI-associated CpGs and the SMFQ score. The linear mixed-effect model is adjusted for sex and age of child, physical activity at the age of 5 years, age of mother at birth, birthweight, mother’s pre-pregnancy BMI, average weekly family income at 7, mother’s highest education qualification, CD4T, Bcell, CD8T, Gran, Mono, NK as fixed effect and chip and position bead array as random effects. The red dots are the CpGs with FDR<0.05. The vertical black dashed line indicates the rate ratio at 1. The vertical axis is the significance (*p*-value) on a log10 scale, and the horizontal axis is the beta estimate.

We next explored the associations of these CpGs with the SMFQ score at 11 years old. Starting from the look-up list of 483 CpGs plus the four CpGs identified in the genome wide EWAS, we analyzed associations with SMFQ score using multiple adjusted Poisson models. Sixty-eight of these CpGs were associated with SMFQ after 5% FDR correction on included look-up CpGs (Supporting Information S2, Supplementary Table S10, Figure 5). Four of these (cg04878366, cg25487405, cg09152259, and cg18548288) had a *p*-value lower than 1.0 × 10^−6^, which is generally considered a suggestive association in genome-wide epigenetic studies (Duggal et al., 2008).

Then, we selected those CpGs associated with 1) BMI and 2) SMFQ in the ALSPAC cohort to further investigate their potential mediation role between obesity at age seven and SMQF at age 11. Ten CpGs remained for mediation analysis (Table 4), after filtering those that had a look-up *p*-value of *p* < 0.05 with BMI and an association with SMFQ of FDR-corrected *p* < 0.05, However, only four of these CpGs–cg17128312, cg25435714, cg26224499, and cg26687842—had matching directions of association (i.e., both hypomethylated or hypermethylated with both BMI and SMFQ score) that may indicate a mediation role.

**TABLE 4:** CpGs simultaneous significantly associated (*p* < 0.05) with BMI at 7 years and (FDR 5%) SMFQ at 11 years.

CpG	Chromosome	Position	UCSC^a^ RefGene name	UCSC RefGene group	Association with BMI at 7 years		Association with SMFQ at 11 years	
					Beta	*p*-value	Rate ratio	*p*-value
cg03431111	chr11	62621406	SNORD30; SNORD22; SNORD29; SNORD31; SNHG1	TSS1500; Body	0.017	1.06E-03	0.685	2.47E-04
cg09664445	chr17	2612406	KIAA0664	5′UTR	0.010	3.59E-02	0.668	4.57E-04
cg11969813	chr17	79816559	P4HB	Body	0.017	1.01E-02	0.697	3.20E-05
cg13781414	chr9	138951648	NACC2	5′UTR	0.010	3.72E-02	0.668	3.90E-04
cg17128312	chr19	12806824	FBXW9	Body	-0.018	4.20E-04	0.720	2.20E-03
cg18219,562	chr17	41773643			0.016	8.33E-03	0.778	3.76E-03
cg21486834	chr17	74477542	RHBDF2; RHBDF2	Body	0.012	7.49E-03	0.643	2.56E-04
cg25435714	chr7	157083381			0.012	4.57E-02	1.308	2.90E-03
cg26224499	chr19	8008578	TIMM44	TSS200	0.015	1.46E-07	1.550	5.31E-04
cg26687842	chr13	41055491	LOC646982	TSS1500	-0.009	3.74E-02	0.643	2.79E-04

a *UCSC:* uniersity of california, Santa Cruz.

### Mediation of obesity and mental health by CpGs

**FIGURE 6:**
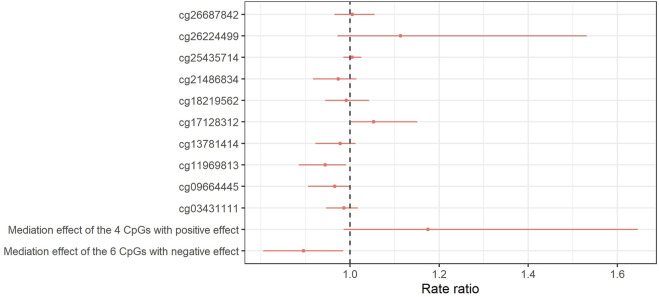
Forest plot of the natural indirect effect of the mediation analysis for the relation between obesity at 7 years and SMFQ score at 11 years as mediated by 10 CpGs simultaneously associated (*p* < 0.05) with BMI and (FDR 5%) SQFM at 11 years. Red dots and bars show rate ratio and 95% CI for natural directed effect, respectively. The model is adjusted for sex, age of mother at birth, birthweight, mother’s pre-pregnancy BMI, average weekly family income at 7, mother’s highest education qualification. 95% CI was calculated by bootstrapping with 1,000 replications.

The natural indirect effects of these CpGs (mediating pathways) are shown in Figure 6. The mediation analysis indicated that the effect of obesity on SMFQ scores at age 11 may be mediated by cg17128312 (RR: 1.05, 95% CI: 1.01, 1.13, PM: 27%). There was no evidence for mediation by cg26687842 (RR: 1.01, 95% CI: 0.97, 1.05, PM: 3%) or by cg25435714 (RR: 1.00, 95% CI: 0.98, 1.02, and PM: 2%) (Supporting Information S2, Supplementary Table S11). On the other hand, there was evidence of a negative mediation (suppression) by cg1196813 (RR: 0.94, 95% CI: 0.88, 097, PM: 30%).

### Methylation risk score

**FIGURE 7:**
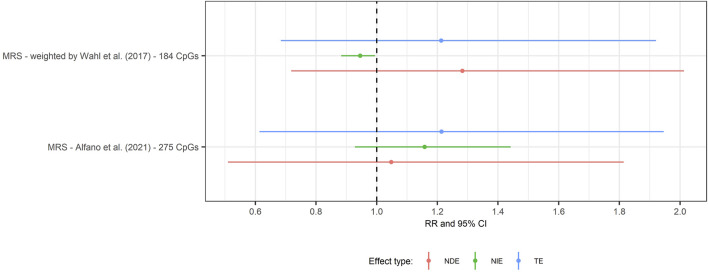
Forest plot of the mediation analysis by methylation risk scores at age 7 years of obesity at 7 years and SMFQ score at 11 years, follow-up. Rate ratio and 95% CI(bars) for natural direct effect (red line), natural indirect effect (green line) and total effect (blue line) from single mediation analysis of obesity at 7 years on SMFQ through MRS. Rate ratio refers to MRS 1) based on Wahl et al. (2017) study (Supporting Information S1, Supplementary Table S1) and 2) based on 275 CpGs associated with child adiposity (Alfano et al., 2021) (Supporting Information S1, Supplementary Table S2).

To combine information across loci, we calculated two weighted MRS: 1) the MRS proposed by Wahl et al. (2017) and 2) based on CpGs reported by (Alfano et al., 2021). The mean square error of the MRS based on Wahl et al. (2017) was 1.95 and the MRS based on CpGs reported by (Alfano et al., 2021) was 0.82, respectively. We did not observe evidence of mediation for either MRS (Figure 7).

### Two-sample MR analysis of molecular features and depression

Following the principle of triangulation (Lawlor et al., 2016), we applied two-sample MR based on publicly available summary statistics on candidate metabolites to test causal associations with depression. None of the tested metabolites showed significant associations at *p* < 0.05. However, again the strongest effects were observed for tyrosine, with one unit increase in tyrosine associated with 0.13 great log odds of depression at *p* = 0.1, estimated using the inverse-variance weighted method (Table 5). Results estimated with MR-Egger and weighted median were similar and shown in Supplementary Tables S1, S2 (Supporting Information S1), respectively. MR-Egger tests did not indicate the presence of pleiotropy. No evidence of association was observed for the other metabolites tested. We were unable to find genetic instruments for conjugated linoleic acid.

**TABLE 5:** Two-sample summary statistics Mendelian Randomisation (inverse-variance weighted method) between candidate metabolites and depression. Beta estimate represents the change in the log odds of depression per unit increase in metabolites.

Metabolite	Number of SNPs	Beta estimates	Standard error	*p*-value
Tyrosine	21	0.13	0.08	0.10
Leucine	13	0.11	0.12	0.36
Apolipoprotein B	34	−0.07	0.07	0.31
Triglycerides in small LDL	47	−0.01	0.06	0.81
Phospholipids in medium LDL	33	−0.08	0.07	0.23
Cholesteryl esters in IDL	43	−0.09	0.07	0.21
Cholesteryl esters to total lipids ratio in large LDL	27	−0.12	0.09	0.18
Phospholipids in very small VLDL	54	−0.01	0.05	0.88
Total lipids in very small VLDL	49	0.01	0.06	0.87

A limited number of genetic instruments were available for only four CpGs that were associated with SMFQ score in the observational analysis: cg09664445 (1 SNP), cg21486834 (1 SNP), cg25435714 (3 SNPs) and cg26687842 (1 SNP). The results of MR analysis are given in Supplementary Table S3 (Supporting Information S1). All associations were non-significant: the strongest association was with cg21486834 in a consistent direction with the observational analysis at *p* = 0.27.

## Discussion

In this study, we investigated the role of metabolites in blood and DNA methylation in the relationship between obesity and depression in children. We found that the amino acids tyrosine and leucine, and conjugated linoleic acid may mediate the association between obesity at age seven and depression at age 11. While we found less evidence for a role of methylated CpG loci as mediators of the obesity-depression relationship, we identified one CpG, cg17128312, located in the FBXW9 gene, that had a potential mediation effect.

Higher levels of tyrosine (an AAA) and leucine (a BCAA) were associated with obesity, SMFQ, and depression symptoms with consistent effects. Both tyrosine and leucine are widely and consistently associated with childhood obesity (Handakas et al., 2021). A recent study in children showed that high tyrosine levels during life are associated with more internalizing behaviors and negative emotions (Van Vliet et al., 2019). Additionally, tyrosine and the AAA phenylalanine are direct precursors for the synthesis of brain neurons catecholamines (neurotransmitters), including norepinephrine, dopamine, and epinephrine. Studies indicate these neurotransmitters can be manipulated by gut microbiota and vice versa, suggesting an impact on host physiology (Strandwitz, 2018). Animal studies showed that gut microbiota play a causal role in the development of features of depression (Kelly et al., 2016) and anxiety (De Palma et al., 2017), supporting further evidence that there is a modulation of neurotransmission that is likely a route of communication along the gut-brain axis. Plasma AAAs, which are directly modulated by the gut microbiota (Handakas et al., 2021) could therefore influence later propensity for mental health disorders through metabolic processes of gut and brain interaction. BCAAs, including leucine, play an important role in the activation of the mammalian target of rapamycin (mTor) pathway–a pathway involved in the control of cell growth and proliferation–and has been associated with a short-term decrease in depressive symptoms (Baranyi et al., 2016). Additionally, Orešič et al. (2011) found that elevated levels of BCAAs were associated with schizophrenia. BCAAs may compete with AAAs for transport across the brain-blood barriers (Fernstrom, 2005; Gruenbaum et al., 2019), and their increase may lead to concentration decreases in neurotransmitters derived from the AAAs, including tryptophan, in the brain. However, evidence produced in this study contrasts the findings from Baranyi et al., 2016, who observed a reverse association of blood plasma BCAAs in adult patients with depression in their cross-sectional study (Baranyi et al., 2016).

Conjugated linoleic acid (CLA) is a positional isomer of linoleic acid where the two double bonds are conjugated (i.e., separated by a single bond) and may be either in cis- or trans-configurations, with 28 possible isomeric forms (Yang et al., 2015). We found evidence for a mediating role of CLA between obesity and mental health but not for linoleic acid itself. There is mixed evidence for the role of CLA in health, with both athero-protective (Bruen et al., 2017) and pro-inflammatory effects (Mazidi et al., 2017) reported. The effects on inflammation, which may underlie effects on mood, are thought to result from a reduction in the arachidonate pool, leading to a reduced production of downstream eicosanoid products, which modulate cytokine production (Yang et al., 2015). The mixed health effects reported for CLA may be due to differences between isomeric forms (Yang et al., 2015), but we were unable to distinguish these with the NMR analytical platform employed. We also found LDL measures to be associated with both obesity and SMFQ scores, mainly direct associations. Perturbations in lipid metabolism, and lipoprotein concentrations are typical symptoms of childhood obesity (Daniels, 2011; Lamb et al., 2011). Beasley et al. (2005) suggested that cholesterol-mediated alterations in nerve terminal structure and function regulate the relationship between low cholesterol and depression pathogenesis. However, Persons and Fiedorowicz (2016) conducted a meta-analysis on the relationship between depression and serum LDL, and observed a U-shaped relationship between the two, suggesting that both high and low levels of serum LDL are associated with an increased risk of depression. However, despite the associations observed in this study, we did not observe evidence that LDL measures may have a mediating role.

This study showed little evidence for a mediating role of differentially methylated CpGs. We found evidence that methylation cg17128312 had a potential mediation effect. cg17128312 is located in the FBXW9 gene, one of a few evolutionarily conserved F-box proteins. FBXW9 is widely expressed in the nervous system (Gu et al., 1996) and is involved in the promotion of neurotransmitter release from GABAergic motor neurons (Sun et al., 2013), presenting a plausible mechanism related to mood regulation. Although this CpG was identified through the EWAS approach and therefore strongly associated with obesity in this study, it was not among the 483 CpGs identified as putatively associated with body mass in the literature review (Alfano et al., 2021) or the study of (Wahl et al., 2017), suggesting that while it may contribute to poorer mental health among ALSPAC children with obesity, this relationship is unlikely to be generalizable to other populations. To date, few CpGs have been consistently associated with body mass in children (Vehmeijer et al., 2020; Alfano et al., 2021), in contrast to adults (Wahl et al., 2017) and indeed metabolites (Handakas et al., 2021). This lack of consistency may be related to the length of time in an obese state, which is shorter for children (Vehmeijer et al., 2020).

DNA methylation at four CpGs (cg07462932, cg07462932, cg07462932, and cg26224499) was associated with BMI at an FDR of *p* < 1.06 × 10–7. Additionally, in a look-up analysis on probes previously associated with childhood BMI and obesity, we found that 10 cpgs were associated with both BMI and SMFQ scores, including cg25435714 and cg13781414. Somineni et al. (2019) applied an EWAS analysis in paediatric patients and showed that cg25435714 and cg13781414 were associated with Crohn’s disease (CD), an inflammatory bowel disease, associated with a reduction of faecal microbial diversity (Pascal et al., 2017). A range of studies demonstrated the potential association between obesity and mental disorders and the role of dysbiosis in the gut-brain (Ligezka et al., 2021) and this dysbiosis can alter the functionalities of the central nervous system, behavior and cognitive function (Carding et al., 2015). Studies have shown that bioactive nutrients and gut microbiota can alter either DNA methylation or signatures through a variety of mechanisms (Tateishi et al., 2009; Lester et al., 2013; Alfano et al., 2021). Hence, microbes within the human gut can influence inflammatory cytokines and the production of antimicrobial peptides, affecting the epigenome through their involvement in generating short-chain fatty acids, vitamin synthesis, and nutrient absorption (Alam et al., 2017).

The strength of our study includes both plasma blood metabolome and epigenome from childhood and a large cohort, enabling assessment of the mood disorder at preadolescence and thus limiting reverse causality. The lack of full assessment by trained psychologists is a weakness, although we used the SMFQ that has been developed to enhance epidemiological use (Thapar and McGuffin, 1998) and has demonstrated significant discrimination of depression in several validation studies (Thapar and McGuffin, 1998; Rhew et al., 2010). We included several sociodemographic and clinical factors in our analysis. However, we did not have complete data related to child nutrition that could be linked to both the metabolome and epigenome in life. Nevertheless, we used family socioeconomic factors and maternal clinical factors such as BMI that can reflect general patterns of family nutrition (Williams et al., 2017) and physical activity (Lampinen et al., 2017). Additionally, we used the BMI reference curves for the United Kingdom to define obesity as this BMI chart provides a more detailed monitoring of both the size and shape of the United Kingdom child population. We examined the mediation role of a range of metabolites on the effect of obesity on mood-related outcomes using both single metabolites and an MRS which further revealed the potential mediation role of the metabolic traits in central biological mechanisms. Although the temporal nature of the study design tested the role of obesity-associated molecular features on subsequent depression, we lacked data on mental health at earlier ages to test whether these features may be associated with depression already present at earlier ages (downstream effects). However, the study population had a limited number of cases of depression (less than 5% of the total population), which limited statistical power, particularly for the epigenetic and stratified analyses. For this reason, we also analyzed the SMFQ score using a Poisson regression and used a bootstrapping approach in the mediation analysis. Hence, our results suggest a potential directionality of the effects, encouraging the conduct of further observational and mechanistic studies (Hernán, 2021). Future studies, with gut-microbiota and high-quality dietary data available, should explore the role of nutrition and the mediating role of gut-bacteria in the central nervous system.

In conclusion, we identified three metabolites, tyrosine, leucine and conjugated linoleic acid and methylation at one CpG site that may potentially mediate the association between obesity and later depression in children. Potential biological plausible mechanisms involving these molecular features include regulation and production of neurotransmitters, inflammation, and modulation of the gut microbiome. These results require replication in further well-powered observational and mechanistic studies.

## Data Availability

The data used for this submission can be made available on request to the ALSPAC Executive. The ALSPAC data management plan describes in detail the policy regarding data sharing, which is done through a system of managed open access. Full instructions for applying for data access can be found here: http://www.bristol.ac.uk/alspac/researchers/access/.
