# Supplementary material for: Molecular mediators of the association between child obesity and mental health
Source: Front Genet. 2022 Aug 31;13:947591. doi: 10.3389/fgene.2022.947591 (PMC9473726; doi:10.3389/fgene.2022.947591)
Supplement: Supplementary file 1 [file DataSheet1.docx]

# Molecular mediators of the association between child obesity and mental health

Evangelos Handakas^1^, Yiwen Xu^2^, Alexa Blair Segal^2^, Maria Carmen Huerta^2^, Kirsty Bowman^3,4^, D Howe^3,4^, Oliver Robinson^1^

^1^Μedical Research Council Centre for Environment and Health, Imperial College London, London, United Kingdom

^2^Centre for Health Economics & Policy Innovation, Department of Economics & Public Policy, Imperial College Business School, South Kensington Campus, London, UK

^3^MRC Integrative Epidemiology Unit at the University of Bristol, Bristol, UK

^4^Population Health Sciences, University of Bristol, Bristol, UK

* Corresponding Author: [o.robinson@imperial.ac.uk](mailto:o.robinson@imperial.ac.uk)

# Supporting information

Table of contents

[Molecular mediators of the association between child obesity and mental health 1](#_Toc105858673)

[Supporting information 1](#_Toc105858674)

[Study population 2](#_Toc105858675)

[Mediation analysis 3](#_Toc105858676)

[Statistical software 6](#_Toc105858677)

[Mendelian Randomisation 7](#_Toc105858678)

[Imputation Analysis 9](#_Toc105858679)

[References 9](#_Toc105858680)

List of figures and tables

[Figure S1 Flow chart of the ALSPAC study population selection based on the inclusion and exclusion criteria. 3](#_Toc107066070)

[Figure S2 Forest plot of mediation analysis for SMFQ score across multiple metabolic compounds for total population and stratified by sex. 4](#_Toc107066071)

[Figure S3 Forest plot of mediation analysis for depression symptoms (SMFQ>7 ) across multiple metabolic compounds for total population and stratified by sex.. 5](#_Toc107066072)

[Figure S4 Forest plot of mediation analysis for depression (SMFQ>11) across multiple metabolic compounds for total population and stratified by sex.. 6](#_Toc107066073)

[Figure S5 Manhattan Plot of EWAS results.. 7](#_Toc107066074)

[Table S1 Two-sample summary statistics Mendelian Randomisation, using the MR-Egger method, between candidate metabolites and depression.. 8](#_Toc107066075)

[Table S2 Two-sample summary statistics Mendelian Randomisation, using weighted median method, between candidate metabolites and depression. 8](#_Toc107066076)

[Table S3 Two-sample summary statistics Mendelian Randomisation between candidate metabolites and depression. 8](#_Toc107066077)

[Figure S6 A) Histogram of raw data. missing levels and B) frequency of missing patterns. 10](#_Toc107066078)

# Study population

Individuals were considered eligible for the analysis if they attended the food recoding campaign had 50% covariates available and had attended a clinical assessment at the age of 7 years. Starting with the 15665 individuals who were participated in the ALSPAC cohort, 7785 individuals attended the clinical assessment at the age of 7 years. These individuals were used for imputing the selected covariates. Then, we excluded the population without Nuclear Magnetic Resonance(NMR) data at the age of 7 years and without Short Mood Feeling questionnaires at the age of 11 years. Finally, we included in the metabolome wide association studies (MWAS) and in the mediation analysis 4018 individuals whose SMDQ and metabolomics data were available. Finally, we carried out an epigenome wide association studies (EWAS) and a mediation analysis in 768 individuals whose epigenetic data was available (Figure S1).


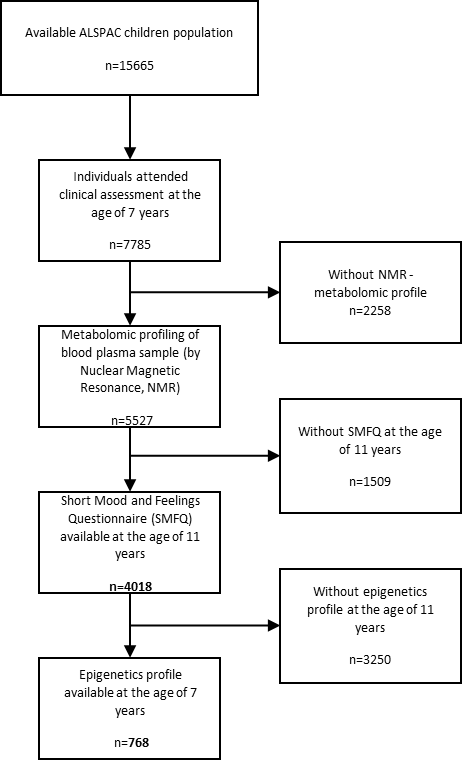


Figure S1 Flow chart of the ALSPAC study population selection based on the inclusion and exclusion criteria.

# Mediation analysis by sex


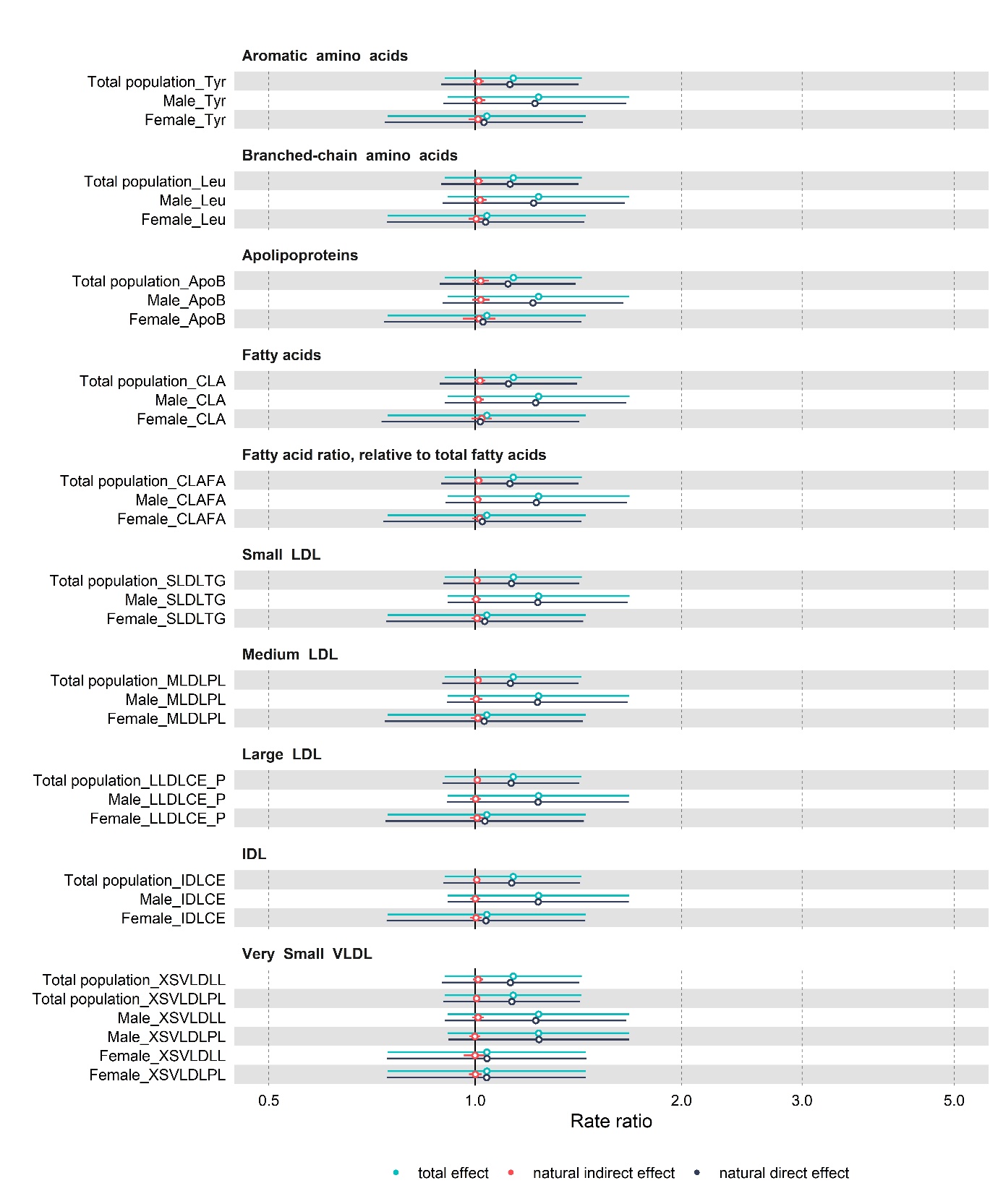


Figure S2 Forest plot of mediation analysis for SMFQ score across multiple metabolic compounds for total population and stratified by sex. Models are adjusted for sex and age of child, physical activity at the age of 5 years, age of mother at birth, birthweight, mother’s pre-pregnancy BMI, average weekly family income at 7, mother’s highest education qualification. Rate ratio is presented as dot. Bars show 95% confidence intervals calculated based on bootstrapping approach (1000 replications).


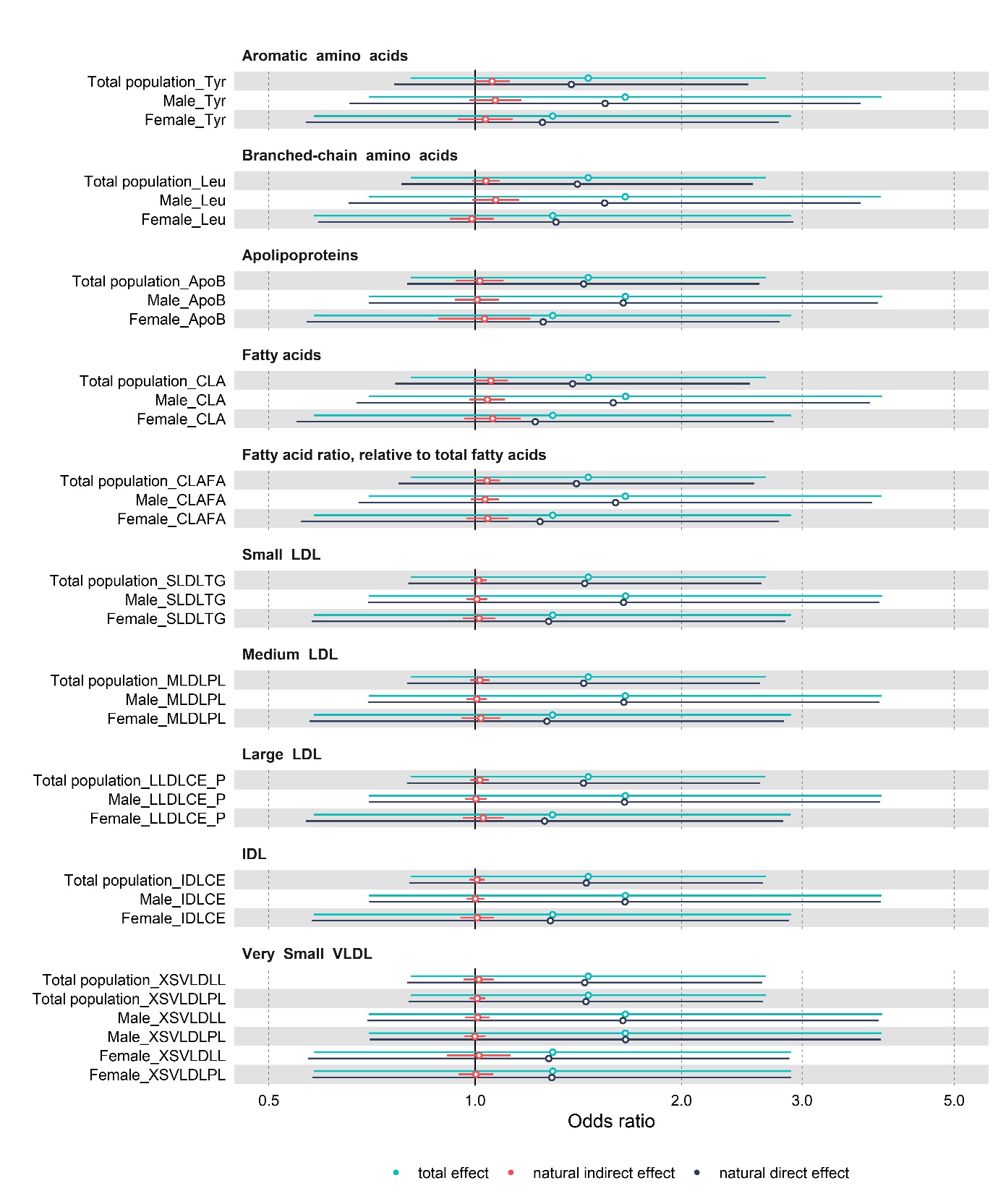


Figure S3 Forest plot of mediation analysis for depression symptoms (SMFQ>7 ) across multiple metabolic compounds for total population and stratified by sex. Models are adjusted for sex and age of child, physical activity at the age of 5 years, age of mother at birth, birthweight, mother’s pre-pregnancy BMI, average weekly family income at 7, mother’s highest education qualification. Odds ratio is presented as dot. Bars show 95% confidence intervals calculated based on bootstrapping approach (1000 replications).


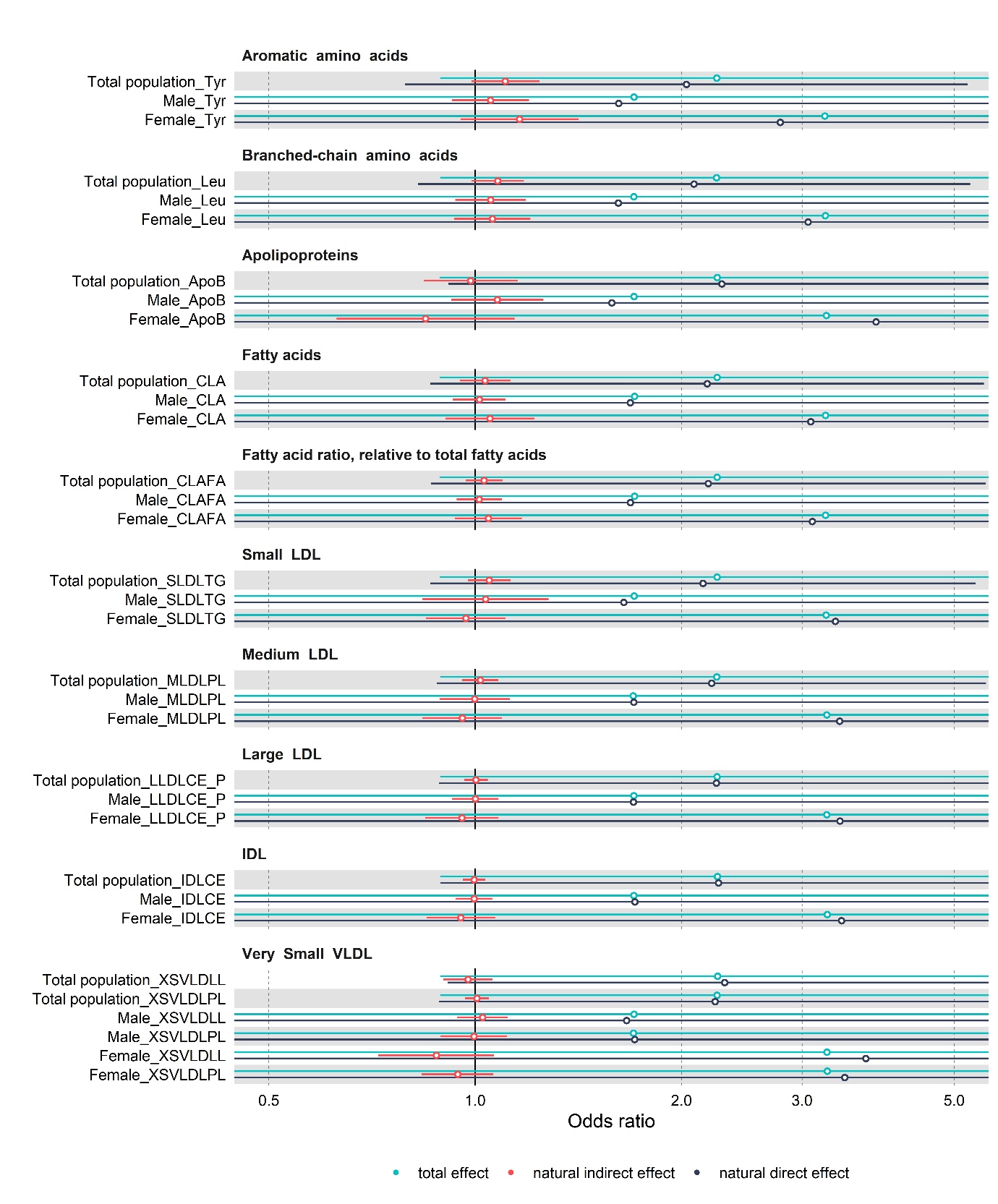


Figure S4 Forest plot of mediation analysis for depression (SMFQ>11) across multiple metabolic compounds for total population and stratified by sex. Models are adjusted for sex and age of child, physical activity at the age of 5 years, age of mother at birth, birthweight, mother’s pre-pregnancy BMI, average weekly family income at 7, mother’s highest education qualification. Odds ratio is presented as dot. Bars show 95% confidence intervals calculated based on bootstrapping approach (1000 replications).


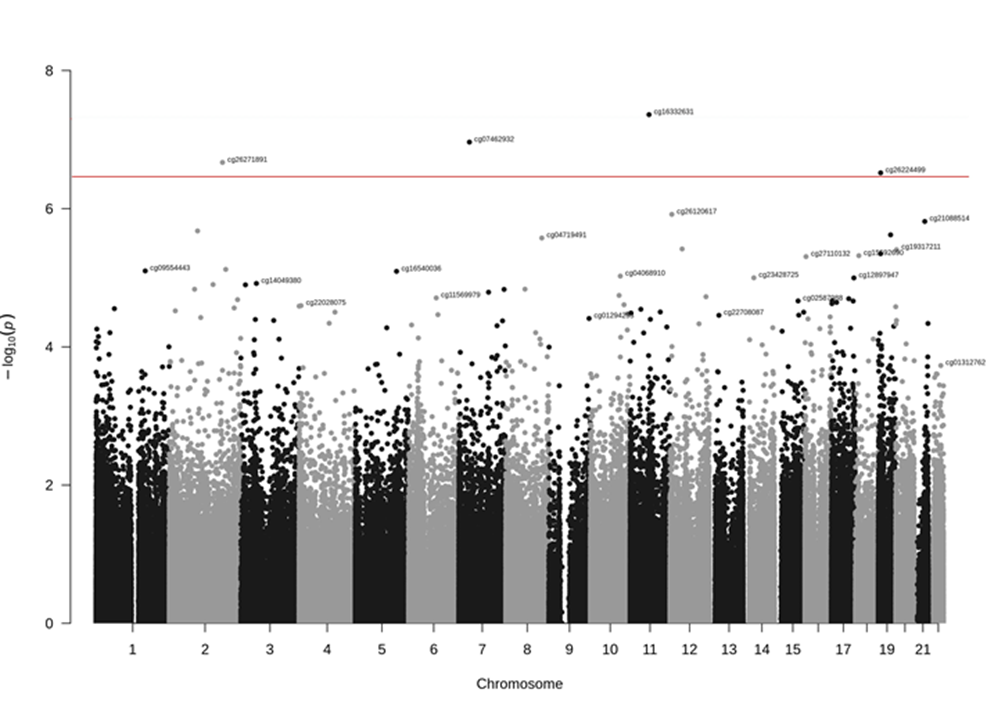


Figure S5 Manhattan Plot of EWAS results. The Manhattan plot depicts the significance of association with BMI as the negative logarithm of the p-value (-log(p-value)) versus the chromosomal location (chromosomes) for each of the ~450 K DNA-methylation probes. The model is adjusted for age, sex, CD4T, Bcell, CD8T, Gran, Mono, NK as fixed effect and chip and position bead array as random effect. The red line marks the threshold for epigenome-wide significance after applying FDR threshold of 5%. The vertical axis is the chromosomal position and the horizontal axis is the significance on a −log10 scale.

# Statistical software

The statistical analyses were performed using R (‘The R Project for Statistical Computing’) software environment (v3.5.2). The mediation analysis was carried out using the medflex package (Steen et al., 2017). ggplot (Wickham, 2016) and circlize (Gu et al., 2014) packages were used for plotting the results. Additionally, the lme4 package was used for mixed-effect regression models (Bates et al., 2014). Multiple mixed-effect logistic and linear regression analysis were carried out using lme4 R package (Bates et al., 2014). Metabolic risk score (MRS) were calculated using caret R package(Kuhn, 2008). We calculated age- and sex-specific Z-scores for child  body mass based on SITAR R package (Cole and Cole, 2020). Shell scripting and doParallel R package were combined for running the analysis in a High-Performance Computer (HPC) environment. The two-sample MR was conducted using the TwoSample R package (Hemani et al., 2018). The imputation analysis of the model covariates and metabolomic data was carried out using the package mice (Buuren and Groothuis-Oudshoorn, 2010) and missForest R package (Stekhoven and Bühlmann, 2012), respectively.

# Mendelian Randomisation

Table S1 Two-sample summary statistics Mendelian Randomisation, using the MR-Egger method, between candidate metabolites and depression. Beta (slope) estimate represents the change in the log odds of depression per unit increase in metabolites.

| Metabolite | Number of SNPs | Beta estimates | Standard error | p-value |
| --- | --- | --- | --- | --- |
| Tyrosine | 21 | 0.16 | 0.12 | 0.20 |
| Leucine | 13 | 0.23 | 0.25 | 0.39 |
| Apolipoprotein B | 34 | -0.15 | 0.13 | 0.29 |
| Triglycerides in small LDL | 47 | -0.02 | 0.11 | 0.86 |
| Phospholipids in medium LDL | 33 | -0.15 | 0.14 | 0.26 |
| Cholesteryl esters in IDL | 43 | -0.26 | 0.15 | 0.09 |
| Cholesteryl esters to total lipids ratio in large LDL | 27 | -0.11 | 0.19 | 0.57 |
| Phospholipids in very small VLDL | 54 | 0.02 | 0.09 | 0.86 |
| Total lipids in very small VLDL | 49 | -0.02 | 0.10 | 0.81 |

Table S2 Two-sample summary statistics Mendelian Randomisation, using weighted median method, between candidate metabolites and depression. Beta estimate represents the change in the log odds of depression per unit increase in metabolites.

| Metabolite | Number of SNPs | Beta estimates | Standard error | p-value |
| --- | --- | --- | --- | --- |
| Tyrosine | 21 | 0.16 | 0.10 | 0.12 |
| Leucine | 13 | 0.12 | 0.16 | 0.45 |
| Apolipoprotein B | 34 | 0.01 | 0.10 | 0.96 |
| Triglycerides in small LDL | 47 | -0.01 | 0.08 | 0.90 |
| Phospholipids in medium LDL | 33 | -0.02 | 0.10 | 0.81 |
| Cholesteryl esters in IDL | 43 | -0.13 | 0.10 | 0.20 |
| Cholesteryl esters to total lipids ratio in large LDL | 27 | -0.06 | 0.11 | 0.59 |
| Phospholipids in very small VLDL | 54 | 0.03 | 0.08 | 0.69 |
| Total lipids in very small VLDL | 49 | 0.03 | 0.07 | 0.66 |

Table S3 Two-sample summary statistics Mendelian Randomisation between candidate metabolites and depression. Beta estimate represents the change in the log odds of depression per percentage increase in methylation.

| CpG | method | Number of SNPs | Beta estimates | Standard error | p-value |
| --- | --- | --- | --- | --- | --- |
| cg09664445 | Wald ratio | 1 | -0.00112 | 0.001264 | 0.38 |
| cg21486834 | Wald ratio | 1 | -0.00134 | 0.001213 | 0.27 |
| cg26687842 | Wald ratio | 1 | 0.0012 | 0.00144 | 0.40 |
| cg25435714 | MR Egger | 3 | 0.000371 | 0.000663 | 0.68 |
|  | Weighted median | 3 | 1.01E-05 | 0.000479 | 0.98 |
|  | Inverse variance weighted | 3 | 6.17E-05 | 0.000435 | 0.89 |

# Imputation Analysis

To maximize power and potentially reduce bias in our analysis, we applied a multivariable multiple imputation procedure to impute the missing values of covariates. In this study we imputed the following covariates: maternal education, family income, frequency of going to park or playground, birthweight, maternal pre-pregnancy BMI and maternal age during pregnancy. The missing levels are presented in Figure S6. The results showed that the data consists of individuals without missing data at a level of 65%. Additionally, the levels of missing values of all variables are lower than 20%.


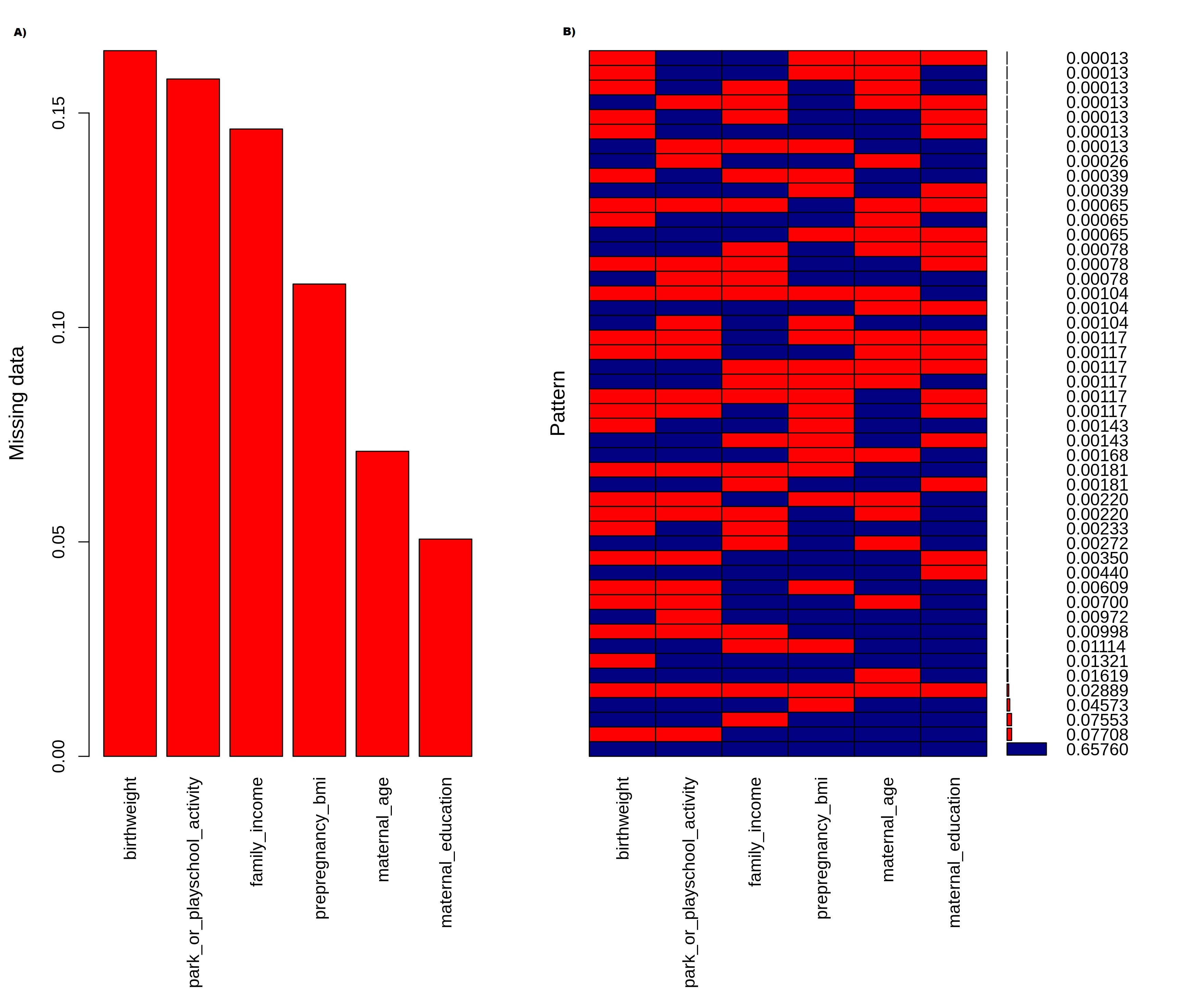


Figure S6 A) Histogram of raw data missing levels and B) frequency of missing patterns.

# References

Bates, D., Maechler, M., Bolker, B., and Walker, S. (2014). lme4: Linear mixed-effects models using Eigen and S4. *R package version* 1(7)**,** 1-23.

Buuren, S.v., and Groothuis-Oudshoorn, K. (2010). mice: Multivariate imputation by chained equations in R. *Journal of statistical software***,** 1-68.

Cole, T., and Cole, M.T. (2020). Package ‘sitar’.

Gu, Z., Gu, L., Eils, R., Schlesner, M., and Brors, B. (2014). circlize implements and enhances circular visualization in R. *Bioinformatics* 30(19)**,** 2811-2812.

Hemani, G., Zheng, J., Elsworth, B., Wade, K.H., Haberland, V., Baird, D., et al. (2018). The MR-Base platform supports systematic causal inference across the human phenome. *elife* 7**,** e34408.

Kuhn, M. (2008). Building predictive models in R using the caret package. *Journal of statistical software* 28**,** 1-26.

Steen, J., Loeys, T., Moerkerke, B., and Vansteelandt, S. (2017). Medflex: an R package for flexible mediation analysis using natural effect models. *Journal of Statistical Software* 76(11).

Stekhoven, D.J., and Bühlmann, P. (2012). MissForest—non-parametric missing value imputation for mixed-type data. *Bioinformatics* 28(1)**,** 112-118.

Wickham, H. (2016). *ggplot2: elegant graphics for data analysis.* Springer.
